# Near-Complete Genome Sequence of Grapevine Fabavirus, a Novel Putative Member of the Genus *Fabavirus*

**DOI:** 10.1128/genomeA.00703-16

**Published:** 2016-07-21

**Authors:** Maher Al Rwahnih, Olufemi J. Alabi, Nathaniel M. Westrick, Deborah Golino, Adib Rowhani

**Affiliations:** a Department of Plant Pathology, University of California, Davis, Davis, California, USA; b Department of Plant Pathology & Microbiology, Texas A&M AgriLife Research and Extension Center, Weslaco, Texas, USA

## Abstract

A novel virus-like sequence from grapevine was identified by Illumina sequencing. The genomic organization was most similar to that of members of the genus *Fabavirus*. Polyproteins RNA-1 and RNA-2 of the virus tentatively named grapevine fabavirus (GFabV) shared 34 to 23% sequence identities with *Broad bean wilt virus 2* (BBWV2), respectively. GFabV was successfully graft transmitted to *Vitis vinifera* cv. Cabernet Franc.

## GENOME ANNOUNCEMENT

The genus *Fabavirus*, family *Secoviridae*, consists of ﬁve viral species: *Broad bean wilt virus 1* (BBWV1), BBWV2, *Gentian mosaic virus*, *Cucurbit mild mosaic virus*, and *Lamium mild mosaic virus*. Fabaviruses infect a wide range of monocotyledonous and dicotyledonous host plants, and they are nonpersistently transmitted by aphids (1). We encountered a novel fabavirus, tentatively named grapevine fabavirus (GFabV), during the characterization of two selections of Japanese table grapes, *Vitis vinifera* cv. Black Beet (BB) and Nagano Purple (NP) introduced to the Foundation Plant Services (FPS), Davis, CA, from South Korea in 2013. To explore the virome of the NP and BB selections, total RNA was extracted from leaf petiole tissue using the MagMAX-96 viral RNA isolation kit, according to the manufacturer’s instructions, and analyzed by high-throughput sequencing (HTS) using the Illumina NextSeq 500 platform. The analysis generated approximately 32 and 34 million Illumina reads (~50 nucleotides [nt] in length) for NP and BB, respectively. Bioinformatics analysis was performed as described by Al Rwahnih et al. (2). tBLASTX analysis from both NP and BB assembled reads revealed two large contigs in each, ranging in size from 3,047 to 5,755 nucleotides and sharing maximum identities with polyproteins RNA-1 (34%), and RNA-2 (23%) of BBWV2 (query coverage, 86 to 90%; *E* value, 0.0 to 3e-4), thus indicating that it is a distinct member of the genus *Fabavirus* based on the established criteria (1). To confirm the HTS results, total RNA was isolated from four vines of each NP and BB cultivar and screened by reverse transcription-PCR (RT-PCR) with two sets of GFabV-specific primer pairs, Ctg438F (5′-AGACAGAATGAGTTGGGTGCTC-3′) and 438R (5′-GCTTACCACGCATATATCAGGC-3′), and Ctg187F (5′-TGGCTTAATTAACCGACCGC-3′) and 187R (5′-AGCCACTTCTAGTGCTCCAAGA-3′). DNA fragments of the expected sizes (642 bp and 628 bp, respectively) obtained from these vines were directly sequenced and determined to be 96 to 99% identical with corresponding sequences generated by HTS. Graft transmissibility of GFabV was confirmed via chip bud grafting from the sources NP and BB vines onto virus-free *Vitis vinifera* cv. Cabernet Franc test plants, as described by Al Rwahnih et al. (2). Successful virus transmission to the test plants was confirmed by RT-PCR using the GFabV-specific primer pairs, as described above. The prevalence of GFabV was investigated by screening a total of 185 grapevine selections from the Foundation Plant Services and the USDA National Clonal Germplasm Repository in Davis, CA, by RT-PCR. These collections were sourced from diverse geographical regions worldwide. In this survey, the occurrence of GFabV was found in two additional selections, one each from India and South Korea. A virus serologically related to BBWV was previously reported from grapevine (3), but its genome sequence was not determined. To the best of our knowledge, the results of this study represent the first report of the occurrence of a virus in the genus *Fabavirus* in North America and the first sequence evidence of its occurrence in grapevines. Field surveys and biological studies are under way to determine the prevalence of GFabV in California, evaluate its potential natural spread, and assess its effect on vine performances and wine quality.

### Nucleotide sequence accession numbers.

The GenBank accession numbers for the sequences of this virus are KX241482 to KX241485.
